# Analysis of characteristic and postexposure practices of occupational blood and body fluid exposures among health care workers in Chinese tertiary hospitals: a retrospective ten-year study

**DOI:** 10.1186/s12879-024-09118-1

**Published:** 2024-02-23

**Authors:** Hong Feng, Xiaoli Mao, Mengqi Li, Hongbo Mao

**Affiliations:** 1 Institute for Zhongnan Hospital of Wuhan University, Wuhan, 430071 China; 2 Wuchang University of Technology, Wuhan, 430065 China; 3 University of Galway, Galway, Ireland

**Keywords:** Healthcare workers, Occupational exposure, Blood-borne pathogens, Postexposure, Practices

## Abstract

**Background:**

Occupational blood and body fluid exposure (OBEs) is a highly concerning global health problem in health facilities. Improper or inadequate post-exposure practices increase the risk of infection with bloodborne pathogens. Understanding risk factors for OBEs and evaluating the post-exposure practices will contribute to healthcare workers’ (HCWs) well-being.

**Methods:**

This study retrospectively synthesized and reviewed the 10-year data (from 2010 to 2020) on OBEs in a tertiary teaching hospital.

**Results:**

A total of 519 HCWs have reported OBEs, increasing yearly from 2010 to 2020. Of these, most were nurses (247 [47.2%]), female (390 [75.1%]), at 23–27 years old (207 [39.9%]). The hepatitis B was the primary bloodborne pathogen exposed to HCWs, with 285 (54.9%) cases, internal medicine was the main exposure site (161 [31.0%]), and sharp injury was the main exposure route (439 [84.6%]). Data analysis shows that there are significant differences between exposure route, exposed pathogens, and exposure site among the different occupational categories (*X*^*2*^ = 14.5, 43.7, 94.3, *all P* < 0.001). 3.3% of HCWs did not take any post-exposure practices. For percutaneous exposure, 4.7% did not rinse the wound, 3.3% did not squeeze out the wound, and 2.3% did not disinfect the wound. In the case of mucosal exposure, 90.4% clean the exposure area immediately.

**Conclusions:**

The data from the past decade underscores the seriousness of current situation of OBEs in Chinese tertiary hospital, particularly among young HCWs, and with hepatitis B as the predominant blood-borne pathogen. This study also identifies HCWs may take incorrect post-exposure practices. It’s crucial in the future to discuss the effectiveness of main groups targeted for focused specialty-specific guidance for the prevention of such accidents, meanwhile, to include blood-borne disease immunity testing in mandatory health check-ups. Additionally, focus on optimizing post-exposure practices, offering significant steps toward prevention of such incidents and reducing infection risks should also be considered in future studies.

## Introduction

The World Health Organization (WHO) estimated that about three million healthcare workers (HCWs) are exposed to occupational blood and body fluid exposures (OBEs) each year [1]. Another study reported the global pooled prevalence of OBEs during career time was 56.6% [2]. These indicated that OBEs was one of the most common occupational accidents [1]. In addition, there is emerging evidence that HCWs had post-traumatic stress disorder, anxiety, and depression after experiencing OBEs, which seriously affect their quality of life and mental health [3–5]. And the high cost of medical care for HCWs who experience accidents significantly impacts hospitals and the state [6, 7]. Excluding the cost of treating infection, the median of the means only caused by needlestick and sharps injury was Int$747 (range, Int$199–Int$1,691) [6]. It is vital to take precautions to reduce the risk of OBEs.

Occupational-related blood-borne pathogens, which refer to the hepatitis B virus (HBV), hepatitis C virus (HCV), human immunodeficiency virus (HIV), treponema pallidum (TP), etc. can be transmitted to human blood and body fluids through eyes, nose, mouth, damaged skin, mucous membranes, or other potentially infectious substances [8]. And the transmission risk is influenced by factors such as injury depth, blood volume, source-patient risk, host immunity, and post-exposure management [9]. Therefore, guideline about the management of OBEs was developed to reduce the risk of infection with bloodborne pathogens, including washing the exposure site with soap and water; and getting post-exposure counseling [10, 11]. It is worth noting that the effectiveness of the guidelines relies on individuals taking appropriate actions and timely reporting after the incident occurs. However, the real prevalence of occupational exposure was under-reporting [12, 13] and many HCWs did not follow the procedure that the guideline recommended [13].

In China, HCWs works for a long time and has excessive paperwork, leaving them exhausted and at high risk of exposure to blood and bodily fluids [14]. For only occupational BBP caused by needlestick and sharps injury (NSI), up to 0.08 per person or 8221 cases per 100,000 HCWs per month was reported in mainland China [15]. Additionally, one survey found that approximately 25% of HCWs did not clean the wound and 30% of HCWs did not apply antiseptics to the wound [16]. Clearly, continuing to explore the risk factors associated with OBEs and incorrect postexposure practices remains of great importance. Therefore, this study investigated the trend of OBEs in HCWs, analyzed the features of OBEs, and evaluated the post-exposure practices over the past ten years in a Chinese tertiary university hospital.

## Methods

This retrospective study was conducted in a Chinese national tertiary care education and research hospital with a capacity of 3,300 beds. The ethics was approved by the hospital’s Clinical Research Ethics Committee(2,023,102 K). We extracted the OBEs data on HCWs from January 2010 to December 2020. Participants with missing necessary information were excluded from this study. The exposed employees were required to fill out a standard form including name, age, gender, department, time and place of exposure, locations of exposure, type of exposure, pathogens of exposure, and description of postexposure practices.

The SPSS statistics software V.26.0 was used for data analysis and Microsoft Excel was used to draw the graphics. Frequency counts and percentages were used to express the categorical variables. Mean and standard deviation were used to describe continuous variables. Chi-squared test and Monte Carlo test were used to examine the relationship between different groups, including exposure route, exposure site, and exposure pathogens. *P* value < 0.05 was considered statistically significant.

## Results

**Fig. 1:**
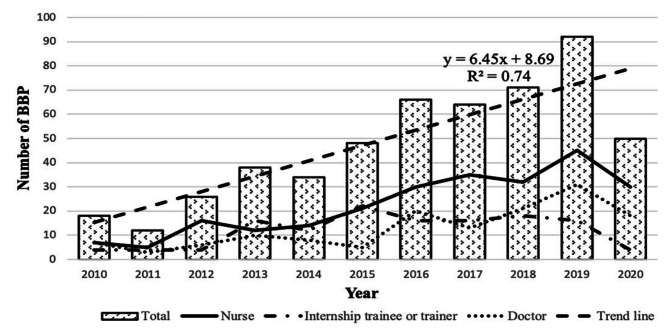
Occupational blood and body fluid exposure of health care workers in a tertiary hospital from 2010 to 2020

A total of 578 HCWs experienced OBEs in the past ten years, excluding 59 sets of data due to incomplete information, 519 HCWs’ data were ultimately included in the analysis. The average number of OBEs each year was 41.05 ± 28.53. Most OBEs were reported in 2019 (92), while the least was reported in 2011 (12). Detailed information is shown in Fig. 1.

There 390 individuals were female and 129 were male. The average age of the HCWs with OBEs was 27.13 ± 7.24 years (18–63 years). There were 245 (47.2%) nurses, 132 (25.4%) internship trainees or trainers, and 142 (27.4%) doctors. The demographic features are shown in Table 1.

**Table 1:** Demographic features of healthcare workers (*N* = 519)

Feature	Frequency	Proportion (%)
Sex		
Female	390	75.1
Male	129	24.9
Age group		
18-22y	142	27.4
23-27y	207	39.9
28-32y	90	17.3
33-37y	42	8.1
≥ 38y	38	7.3
Occupational category		
Nurse	245	47.2
Doctor	142	27.4
Internship trainee or trainer	132	25.4

Most accidents were caused by sharp injury, with 439 (84.6%) cases, followed by blood or body fluid splash, with 80 (15.4%) cases. HBV was the most common exposed pathogen, with 285 (54.9%) cases, followed by HIV, with 64 (12.3%) cases. Data analysis showed that there are significant differences between exposure route, exposed pathogens, and exposure site among the different occupational categories (all variables have *P* < 0.001), (see Table 2).

**Table 2:** Comparison of the characteristics of occupational blood-borne exposure by occupational category of health care workers (*N* = 519)

variable	Nurse, *n* = 245(%)	Internship trainee or trainer, *n* = 132(%)	Doctor, *n* = 142(%)	Frequency(%)	*X* ^2^	*P*
Exposure route					14.5	0.001
Sharp injury	205(83.7)	124(93.9)	110(77.5)	439(84.6)		
Mucocutaneous	40(16.3)	8(6.1)	32(22.5)	80(15.4)		
Exposed pathogens					43.7	< 0.001 ^a^
HBV	134(54.7)	82(62.1)	69(48.6)	285(54.9)		
HCV	24(9.8)	11(8.3)	8(5.6)	43(8.3)		
HIV	30(12.2)	9(6.8)	25(17.6)	64(12.3)		
TP	21(8.6)	14(10.6)	17(12.0)	52(10.0)		
Coinfection	28(11.4)	15(11.4)	18(12.7)	61(11.8)		
Unknown	8(3.3)	1(0.8)	5(3.5)	14(2.7)		
Exposure site					94.3	< 0.001 ^a^
Internal Medicine	86(35.1)	55(41.7)	20(14.1)	161(31.0)		
General Surgery	59(24.1)	48(36.4)	50(35.2)	157(30.3)		
Intensive care unit	41(16.7)	4(3.0)	10(7.0)	55(10.6)		
Operating room	29(11.8)	7(5.3)	14(9.9)	50(9.6)		
ENT	7(2.9)	3(2.3)	19(13.4)	29(5.6)		
Gynecology & Pediatrics Department	13(5.3)	8(6.1)	25(17.6)	46(8.9)		
Auxiliary medical department	2(0.8)	6(4.5)	2(1.4)	10(1.9)		
Other	8(3.3)	1(0.8)	2(1.4)	11(2.1)		

^a^ Monte Carlo test. HCV: hepatitis C virus; HBV: hepatitis B virus; TP: syphilis; HIV: human immunodeficiency virus

After OBEs, seventeen cases (3.3%) did not take any postexposure practice, including 10 cases of percutaneous exposure and 7 cases of mucosal exposure. In addition, among 429 HCWs with exposure wounds, 4.7% did not rinse the wound, 3.3% did not squeeze the wound, and 2.3% did not disinfect the wound. Of the 73 HCWs with mucosal exposure, 66 (90.4%) cleaned the exposure area immediately (see Table 3).

**Table 3:** Comparison of the postexposure conduct by occupational category of health care workers (*N* = 519)

Postexposure practices	Frequency	Proportion (%)
Wound(*n* = 429) squeeze		
Yes	415	96.7
No	14	3.3
rinse		
Yes	409	95.3
No	20	4.7
disinfect		
Yes	419	97.7
No	10	2.3
Mucosa (*n* = 73) rinse		
Yes	66	90.4
No	7	9.6

## Discussion

The current study found that in the past ten years, the total number of HCWs with OBEs has been increasing year by year, and this result is similar to the 546 needlestick injuries reported in a tertiary care hospital in Turkey over nine years [17], while being much higher than the 195 people with occupational exposure in a hospital in Japan within 19 years [18]. The difference may be related to the different regular training on occupational safety precautions and transmission of infectious diseases, limited medical staff, and inadequate supervision by the health administrators. It is worth noting that the trend toward increasing the number of OBEs between 2010 and 2019. In fact, interventions have been undertaken to reduce the incidence of OBEs, such as occupational safety training for all hospital staff and training for students. These interventions encouraged activities such as prescribing oral medications instead of injections when applicable and promoting the use of security equipment, such as safety-engineered needles and sharps. In addition, specialized guidance for high-risk groups has been developed, for example, we emphasized the importance of safe injections among nurses and established safe zones for sharps exchange among surgeons. We also recommended occupational safety education be offered to undergraduate. While some factors, such as hospital expansion, the convenience of informatization, and the simplified reporting process, could not be ruled out. We are unable to determine whether these interventions are effective or to what extent they are effective in this hospital. Numerous previous studies indicate that strategies like occupational safety education and the use of safely-engineered needles and sharps are effective [8, 9, 19]. It is crucial to further investigate the effectiveness of these measures in the future studies. In 2020, the number of accidents decreases, which may be related to the impact of COVID-19.

Consistent with the findings of many studies [20, 21], the results of the current study demonstrated that the main group of OBEs were nurses. It is connected to the higher female ratio among nurses and the nature of the nursing work. Different from Yoko’s study, which reported NSI among trainee nurses was the highest group (44.6%) [17], our study showed that 25.4% of internship trainees or trainers reported OBEs. The internship trainees or trainers in this study are not allowed to work independently, which must be carried out under the teachers’ guidance to reduce the risk of OBEs. Our study showed that OBEs was concentrated among young HCWs. As previous studies indicated that the incidence of OBEs among young HCWs was related to their insufficient work experience and skills [13, 22]. Internal medicine and general surgery were the most common exposure sites with significant differences in the different occupational categories, which is similar to these reports that doctors in general surgery are the group with the highest of occupational exposure in Italy, as well as other Countries [6, 23]. Compared with physicians, surgeons are more likely to be exposed to sharp instruments, such as scalpels, and suture needles. Nurses of internal medicine departments were more often characterized by more invasive procedures, such as the insertion of peripheral vascular catheters and IV therapy. We found that the sharp injury was the main occupational exposure reason, accounting for 84.6%. Previous studies proved that by reducing the use of sharps and promoting protective equipment, the majority of blood-borne occupational exposure may be avoided [19].

According to reports, more than 20 pathogens can transmit diseases through the blood and body fluids of sharps injuries, among which HBV, HCV, and HIV are more dangerous [24]. The study found that more than half of the health workers (54.9%) were exposed to the hepatitis B pathogens, the same as the results in other studies [25]. The number of the hepatitis B cases in China is at a very high level, at approximately 100,000 cases [26]. In China, HBsAb screening for HCWs is not included in the annual checkup, and hepatitis B vaccination is voluntary. Thus, we strongly recommend that HCWs be vaccinated with the hepatitis B vaccine and check the HBsAb regularly. Fourteen cases of blood-borne pathogens pathogen are unknown. It may be related to the fact that the source of exposure cannot be identified due to the secondary sorting of sharps or the centralized sorting of medical equipment, etc. Following the OBEs management in this hospital, the doctor evaluates staff exposure site and makes treatment suggestions, after which a health record is prepared in medical care department to construct a personalized follow-up plan. HBsAg positivity, for example, implies that the HCWs is currently infected with the hepatitis B virus and will be treated as a chronic hepatitis B or HBsAg carrier depending on the liver function findings.

Concerning the postexposure practice, there were 3.3% of HCWs had not undergone any post-exposure practices. Although supervisors have been urging employees to pay attention to post-exposure practices, which have considerable benefits in reducing the risk of infection. We also found that there are some incorrect postexposure practices, including not rinsing the puncture wound immediately (4.7%), expressing blood or fluid from the wound (3.3%), and using antiseptic solution (2.7%). The result is lower than that reported by Nongyao Kasatpibal in Thailand [13]. The differences may be because of the two countries’ guidelines on post-exposure practice. It is generally acknowledged that the efficacy of wound cleaning is in preventing the risk of infection with bloodborne pathogens. However, Chinese guidelines suggest gently squeezing the wound from the proximal to the distal end [11], while squeezing the wound is not recommended in Thai guidelines [13]. In addition, another guideline shows that the application of antiseptics is not contraindicated as no scientific evidence shows that using antiseptics for wound care further reduces the risk of transmitting a bloodborne pathogen [10]. Therefore, there are regional differences in postexposure best practices.

The main limitation of this study is that it was a retrospective study that only focused on the HCWs who reported after occupational exposure. and the data were obtained from a tertiary teaching hospital with limited generalizability. Second, over the past decade, the occupational exposure registration form has been refined to enhance data detail. Early records, however, lack certain specifics on the timing of incidents and work tenure.

## Conclusions

From the data of the past decade, we found that the situation of OBEs is serious in Chinese tertiary hospitals. Young HCWs are the main groups for OBEs, the hepatitis B is the most common blood-borne pathogen, and some incorrect post-exposure practices are also confirmed in this study. It’s necessary in the future to discuss the effectiveness of main groups be targeted for focused specialty-specific guidance for the prevention of such accidents, and to include blood-borne disease immunity testing in mandatory health check-ups. Additionally, future research should focus on optimizing post-exposure practices, offering significant steps towards preventing such incidents and reducing infection risks.

## Data Availability

The data of this study is available from the corresponding author upon reasonable request.

## Abbreviations

HCWs: Health care works
BBP: Blood-borne pathogen
SPSS: Statistical Package for Social Science
HCV: Hepatitis C virus
HBV: Hepatitis B virus
TP: Syphilis
HIV: Human immunodeficiency virus
